# Frailty in Wild-Type Transthyretin Cardiac Amyloidosis: The Tip of the Iceberg

**DOI:** 10.3390/jcm10153415

**Published:** 2021-07-31

**Authors:** Amaury Broussier, Jean Philippe David, Mounira Kharoubi, Silvia Oghina, Lauriane Segaux, Emmanuel Teiger, Marie Laurent, Isabelle Fromentin, Sylvie Bastuji-Garin, Thibaud Damy

**Affiliations:** 1 Univ Paris Est Creteil, INSERM, IMRB, F-94010 Creteil, France; jean-philippe.david@aphp.fr (J.P.D.); lauriane.segaux@aphp.fr (L.S.); marie.laurent@aphp.fr (M.L.); sylvie.bastuji-garin@aphp.fr (S.B.-G.); thibaud.damy@aphp.fr (T.D.); 2 AP-HP, Hopitaux Henri-Mondor/Emile Roux, Department of Geriatrics, F-94456 Limeil-Brevannes, France; isabelle.fromentin@aphp.fr; 3 AP-HP, Hopital Henri-Mondor, Department of Internal Medicine and Geriatrics, F-94010 Creteil, France; 4 AP-HP, Hopital Henri-Mondor, Department of Cardiology, Heart Failure and Amyloidosis Unit, F-94010 Creteil, France; mounira.kharoubi@aphp.fr (M.K.); silvia.oghina@aphp.fr (S.O.); emmanuel.teiger@aphp.fr (E.T.); 5 AP-HP, Hopital Henri-Mondor, Clinical Research Unit (URC Mondor), F-94010 Creteil, France; 6 AP-HP, Hopital Henri-Mondor, Department of Public Health, F-94010 Creteil, France

**Keywords:** cardiac amyloidosis, wild-type TTR amyloidosis (ATTRwt), frailty, heart failure, elderly, geriatric assessment

## Abstract

ATTRwt-CA occurs in elderly patients and leads to severe heart failure. The disease mechanism involves cardiac and extracardiac infiltration by amyloid fibrils. The objectives of this study are to describe the frailty phenotype in patients with ATTRwt-CA and to assess the associations between frailty parameters, the severity of cardiac involvement, and the course of amyloid disease. We used multidimensional geriatric tools to prospectively assess frailty in patients with ATTRwt-CA consulting (in 2018–2019) in the French National Reference Center for Cardiac Amyloidosis. We included 36 patients (35 males; median age: 82 years (76–86). A third of the patients were categorized as NYHA class III or IV, and 39% had an LVEF below 45%. The median serum NTproBNP was 3188 (1341–8883) pg/mL. The median duration of amyloidosis was 146 months (73–216). The frequency of frailty was 50% and 33% according to the physical frailty phenotype and the Short Emergency Geriatric Assessment questionnaire, respectively. Frailty affected a large number of domains, namely autonomy (69%), balance (58%), muscle weakness (74%), malnutrition (39%), dysexecutive syndrome (72%), and depression (49%). The severity of CA was significantly associated with many frailty parameters independently of age. Balance disorders and poor mobility were also significantly associated with a longer course of amyloid disease. Frailty is frequent in patients with ATTRwt-CA. Some frailty parameters were significantly associated with a longer course of amyloid disease and CA severity. Taking into account frailty in the assessment and management of ATTRwt should improve patients’ quality of life.

## 1. Introduction

Amyloidosis is a systemic disease that affect various organs. Wild-type transthyretin cardiac amyloidosis (ATTRwt-CA, also referred to as senile cardiac amyloidosis) is frequently encountered in older adults. ATTRwt-CA is characterized by extracellular deposits of fibrillar transthyretin proteins, produced mainly by the liver, in soft tissue (such as the heart), in the integumentary system, and in the nerves [1,2,3,4].

The pathophysiological impacts of cardiac infiltration have been described in detail [5,6,7]. According to postmortem studies, the prevalence of ATTRwt-CA in adults over the age of 80 years is 25% [8]. This disease accounts for up to 13% of cases of heart failure (HF) with preserved ejection fraction (HFpEF) and left ventricular hypertrophy (≥12 mm) (i.e., around 6% of all cases of HF) [9] and is associated with a poor prognosis.

The role of inflammatory cardiac microenvironment cannot be ignored. Inflammation contributes to the onset and progression of cardiovascular disease. Preclinical and clinical studies have shown that leukotrienes are overexpressed during atherosclerosis, myocardial infarction, and stroke, confirming their central role in the pathophysiology of cardiac damage [10]. Immunotherapies are revolutionizing modern cancer treatments; nevertheless, the real incidence of early and late adverse events associated with immune checkpoint inhibitors are largely unknown [11]. A similar interplay between inflammation and new treatments in ATTRwt CA could be possible and remains to be determined [12] Previous studies emphasized the high frequency of the various types of extracardiac infiltration, which result in disorders such as carpal tunnel syndrome, lumbar spinal stenosis, and hearing loss [13,14,15,16,17]. Indeed, recent studies have shown that hearing loss was more prevalent and more severe in patients with ATTRv amyloidosis than in age-matched controls from the general population. The hearing loss for all frequencies observed in both ATTRv and ATTRwt (in contrast with the high frequencies only in presbycusis) suggests that amyloid deposits infiltrate the structures of the inner and middle ear [18,19]. The health impact of extracardiac amyloid fibril infiltration in older adults has not previously been studied. Therefore, we suspect pathophysiological links between amyloid infiltration and the frailty frequently observed in elderly heart failure patients [20,21].

Frailty is a multidimensional syndrome in which a decreased physiological reserve capacity impairs the body’s stress adaptation mechanisms; hence, this precarious equilibrium can decompensate when a stressful event occurs. Furthermore, frailty increases the likelihood of adverse health outcomes, such as falls, disability, hospitalization, and death [22]. Given the emergence of (expensive) new treatments for ATTRwt-CA, it is important to have accurate information on frailty and its potential impact on patient follow-up [23]. Currently, only one study has investigated the prevalence and prognostic significance of frailty using a global score (Clinical Frailty Scale (CFS)) in cardiac amyloidosis. It found a prevalence of 39% in an ATTR (wt and hereditary) population, and frailty was significantly associated with mortality (*p* < 0.001) and opens the path to studying the impact on frailty domains [24].

We hypothesize that extracardiac involvement in ATTRwt-CA increases the severity of frailty.

The objectives of the present study are (i) to provide a full description of frailty among patients with ATTRwt-CA and (ii) to assess potential associations between frailty parameters on one hand and CA severity and the course of amyloid disease on the other.

## 2. Materials and Methods

### 2.1. Study Design and Participants

**Figure 1:**
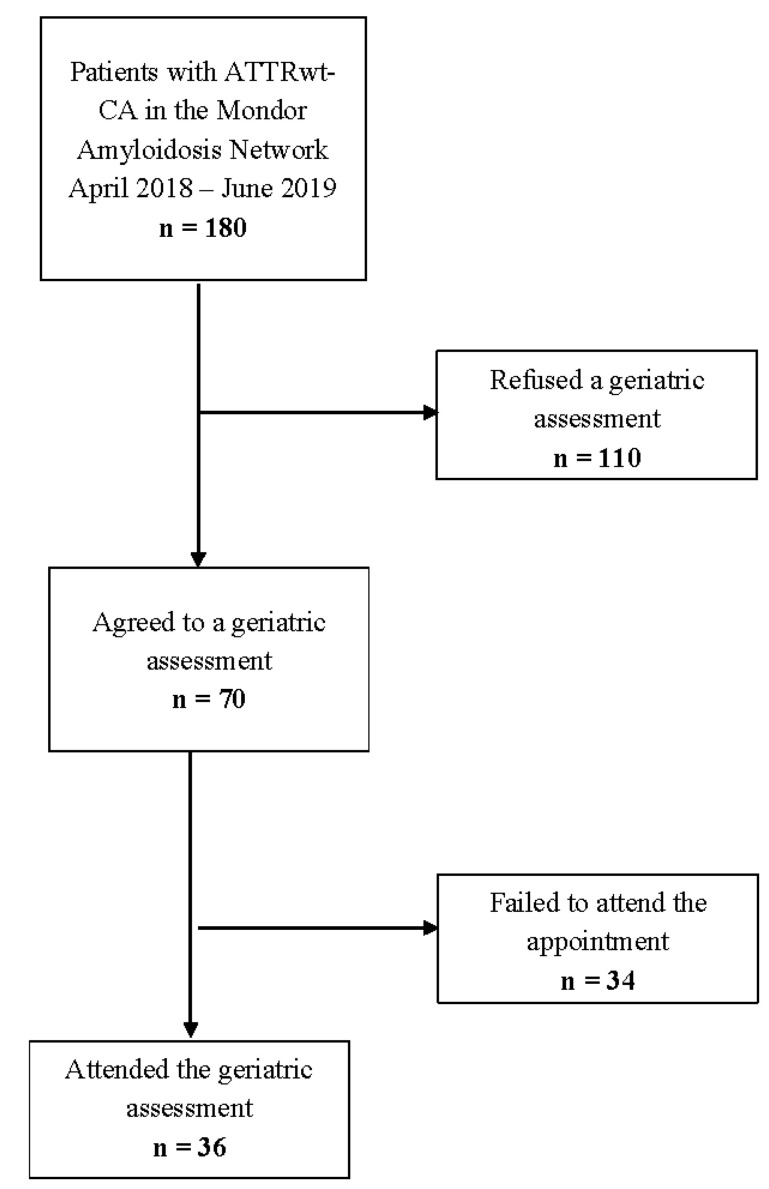
Study flowchart. Consecutive patients with ATTRwt-CA consulting at the French National Reference Center for Cardiac Amyloidosis having agreed to undergo a multidimensional geriatric assessment were prospectively included in the study between April 2018 and June 2019. ATTRwt-CA: wild-type transthyretin cardiac amyloidosis.

We performed a cross-sectional analysis of patients with ATTRwt-CA consulting at the French National Reference Center for Cardiac Amyloidosis (hosted by the Department of Cardiology at Henri-Mondor University Hospital, Creteil, France) between April 2018 and June 2019. Consecutive patients with ATTRwt-CA having agreed to undergo a multidimensional geriatric assessment were prospectively included in the study (Figure 1).

### 2.2. Diagnosis of ATTRwt-CA

The diagnosis of ATTRwt-CA was confirmed by the observation of strong cardiac uptake (visual score ≥ 2) of a bisphosphonate tracer (99mTc-hydroxymethylene diphosphonate) on scintigraphy, and the absence of a TTR gene mutation. Blood and urine samples were analyzed with protein electrophoresis, immunofixation, and a light chain assay. If gammapathy was present, the diagnosis of ATTRwt-CA had to be confirmed by Congo Red staining and TTR immunostaining (in the absence of light chain staining) on an extracardiac and/or endomyocardial tissue biopsy.

### 2.3. Cardiac Assessment

The date of appearance of the first cardiac symptom of amyloidosis (conduction disorder, atrial fibrillation, dyspnea, edema of the lower limbs, cardiac hypertrophy, chest pain, or aortic stenosis) and that of the first extracardiac symptom (carpal tunnel syndrome, lumbar canal stenosis, hearing loss, Dupuytren’s syndrome, or peri-orbital bruising) were recorded. Dyspnea was rated according to the New York Heart Association (NYHA) class. Orthostatic hypotension was defined as a decrease in systolic blood pressure of at least 20 mmHg or a decrease in diastolic blood pressure of at least 10 mmHg upon standing. Blood sample was collected and assayed for NTproBNP and high-sensitivity troponin. All patients underwent standard transthoracic echocardiography (Vivid 7, GE Healthcare, Buc (78), France) and 99mTc-hydroxymethylene diphosphonate cardiac scintigraphy. The left ventricular ejection fraction (LVEF) was calculated using Simpson’s biplane method. The echocardiographic global longitudinal strain (GLS) and the myocardial contraction fraction (MCF) were measured as indices of left ventricle function [25].

### 2.4. Geriatric Assessment

A comprehensive geriatric assessment (CGA) (including physical measurements, performance tests, and a standardized multidimensional clinical evaluation) was performed in the cardiogeriatric unit at Emile Roux University Hospital (Limeil-Brévannes, France). Comorbidities were evaluated on the modified Cumulative Illness Rating Scale [26]. Autonomy was assessed using the Activities of Daily Living (ADL) scale (lack of autonomy: <6/6, i.e., <6 out of 6) and Instrumental Activities of Daily Living (IADL) scale (lack of autonomy: <8/8) [27,28]. Participants were asked about unintentional weight loss over the previous year, and the Mini Nutritional Assessment Scale—Short Form score was recorded (risk of malnutrition: <12/14) [29]. Mobility and muscle strength were assessed with the Short Physical Performance Battery [30], a 6-min walking test [31], gait speed over 10 m (slowness: <1 m/s) [32], and dynamometer-measured maximum dominant-hand grip strength (JAMAR^®^, Sammons Preston, Bolingbrook, IL, USA; weakness: <30 kg in men and <20 kg in women) [33]. Balance was assessed using the one-leg standing test (impaired performance: <5 s) [34] and the self-reported occurrence of non-accidental falls in the previous 12 months. Overall cognitive performance was evaluated using the Mini-Mental State Examination score after adjustment for age and sociocultural level [35]. The five-word screening test was used to assess episodic memory (impairment: <10/10) [36], the seven-point clock-drawing test was used to assess executive and visuospatial functions (impairment: <7/7) [37], and the Frontal Assessment Battery (impairment: <16/18) [38] was used to assess frontal lobe functions. Ten-meter walking test times during concurrent motor and cognitive tasks were recorded. We used the Geriatric Depression Scale to explore mood (risk of depression: ≥5/15) [39]. Sphincter disorders were assessed using the Urinary Symptom Profile questionnaire [40].

Various operational definitions of frailty have been developed; most are based on an assessment of physical frailty alone (such as Fried’s model [22], the most frequently applied) or a multidomain assessment [41]. We used two different tools (one of each type) to estimate the patients’ degree of frailty. First, Fried’s model defines the physical frailty phenotype as robust, pre-frail, or frail by considering loss of bodyweight (>4.5 kg in the previous 12 months), weakness, exhaustion (defined as an answer of “yes” to the question “Did you feel any significant or unusual fatigue over the previous year?”), slowness, and a low level of physical activity [22]. Patients meeting three or more criteria were considered to be frail, those meeting one or two criteria were considered to be pre-frail, and those not meeting any criteria were considered to be robust. Second, we used the Short Emergency Geriatric Assessment (SEGA); this is a simple, validated tool for detecting frailty in elderly subjects as part of a multidimensional approach (not frail: score ≤ 8; frail and very frail: score > 8) [42].

### 2.5. Statistical Analysis

In order to estimate the severity of CA, patients were stratified according to the Gillmore staging system. Stage I was defined as a serum NT-proBNP level ≤ 3000 ng/L and an eGFR ≥ 45 mL/min, stage III was defined as a serum NT-proBNP level > 3000 ng/L and an eGFR < 45 mL/min, and the remaining patients were classified as stage II [43]. Given the small patient population, stages I and II were pooled for comparison with stage III. The duration of amyloidosis was estimated as the time (in months) between the first symptom of amyloidosis (whether cardiac or extracardiac) and the geriatric assessment. The dates of onset for cardiac and extracardiac symptoms were recorded during the patient’s first consultation at the French Referral Center for Cardiac Amyloidosis.

We compared the frailty variables as a function of the severity of CA (the Gillmore stage) by using Fisher’s test or the Kruskal–Wallis test, where appropriate. For variables that yielded *p* values below 0.20, the corresponding odds ratio (OR) (95% confidence interval (CI)) was estimated using exact logistic regression analyses. Age-adjusted ORs were also estimated. We then investigated the potential relationships between the course of the disease and frailty parameters using the Kruskal–Wallis test or Spearman’s rank correlation test, where appropriate. Age-adjusted logistic regression and quantile regression models were also built.

The quantitative and qualitative variables were described as the median (interquartile range) and the frequency (percentage), respectively. All statistical analyses were performed with STATA software (V14.1, StataCorp, College Station, TX, USA). The threshold for statistical significance was set at *p* ≤ 0.05.

## 3. Results

### 3.1. Characteristics of the Study Population

During the study period, 36 of the 180 patients with ATTRwt-CA included in the Henri-Mondor Amyloidosis Network underwent a geriatric assessment and were thus enrolled (110 refused, and 34 agreed but did not attend the appointment) (Figure 1). The only significant difference (*p* = 0.05) was a higher proportion of males in the included group (Supplemental Table S1).

The baseline characteristics of the study population are summarized in Table 1. The median (interquartile range) age was 82 (76–86), and there were 35 men (97%). About a third of the patients were scored as NYHA class III or IV, 39% had left ventricular systolic dysfunction (LVEF < 45%), 58% had atrial fibrillation, and the median serum NTproBNP level was 3188 pg/mL (1341–8883). The median GLS was low: −9.9% (−7.8; −12.5). The median course of amyloidosis disease was 146 months (73–216). The median number of medications was 8 (7–10); on inclusion, none of the patients had been treated with tafamidis. The other treatments complied with the guidelines (no beta blockers, low doses of ACE inhibitors, and ARBs) applied in the French Referral Center for Cardiac Amyloidosis.

### 3.2. Frequency of Organ Impairment and Frailty

**Figure 2:**
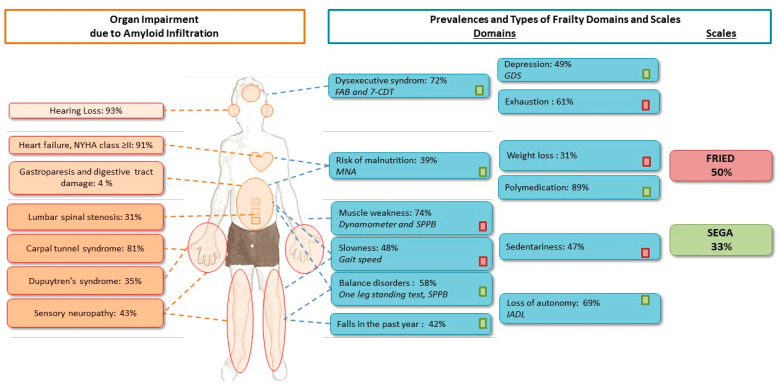
Frequency of frailty, and the relationships between frailty and organ impairment in patients with A-TTRwt-CA. The prevalence of frailty among patients with ATTRwt-CA can be explained by the amyloid fibril infiltration of various organs and tissues, particularly the heart, integumentary system, and nerves. FAB: frontal assessment battery, GDS: geriatric depression scale, IADL: instrumental activity of daily living, MNA: mini nutritional assessment, NYHA: New York heart association, SEGA: short emergency geriatric assessment SPPB: short physical performance battery, 7-CDT: 7-clock drawing test.

The most frequent extracardiac symptoms associated with amyloidosis were hearing loss (93%), carpal tunnel syndrome (CTS) (81%) (79% of the CTS were bilateral; 75% of the CTS, whether uni- or bilateral, had a history of CTS surgery), sensory neuropathy (43%), Dupuytren’s syndrome (35%), and lumbar spinal stenosis (31%) (Figure 2). According to the physical frailty phenotype, 8% of the patients were robust and 50% were frail. According to the SEGA, 33% were frail. Frailty affected a large number of domains, especially cognition, mood, polypharmacy, mobility, balance, autonomy, and sphincter disorders (Table 2 and Figure 2).

### 3.3. Associations between Frailty Parameters and CA Severity (According to the Gillmore Stage)

Stage III patients had a higher NYHA class (*p* = 0.02), a lower LVEF (*p* = 0.04), and a lower MCF (*p* = 0.04) than the stage I and II patients (Supplemental Table S2). Furthermore, stage III patients were significantly older; more likely to be frail (according to the SEGA) and at risk of malnutrition; and more likely to have balance disorders (according to the one-leg standing test), lower gait speed, lower physical performance (according to the Short Physical Performance Battery), lower limb weakness (according to the sit-to-stand test), loss of autonomy, lower performance in cognitive dual task, memory complaints, overactive bladder, and dysuria (Table 2). A nonsignificant trends towards an association with CA severity was observed for executive function impairment (according to the clock-drawing test). After adjustment for age, CA severity was still significantly associated with frailty (according to the SEGA), loss of autonomy, lower limb muscle weakness, balance disorders, memory complaints, and overactive bladder. Nonsignificant trends towards an association were observed for the risk of malnutrition and lower physical performance.

### 3.4. The Relationship between Frailty Parameters and the Course of Amyloid Disease

The course of amyloid disease was not significantly associated with CA severity, as shown in Table 2. In age-adjusted analyses, a longer course of amyloid disease was significantly associated with balance disorders and a slower gait speed in cognitive and motor dual tasks (Supplemental Table S3). Nonsignificant trends towards an association with a longer course of amyloid disease were observed for frailty (according to the SEGA), loss of autonomy, and lower limb muscle weakness.

## 4. Discussion

Frailty in older adults with ATTRwt-CA has not been extensively studied. To the best of our knowledge, the present study is the first to have provided a full clinical description of frailty in patients with ATTRwt and highlighted its association with cardiac and extracardiac infiltration. Our results highlighted a high frequency of frailty—from 33% to 50%, depending on the tool used. We observed impairments in many domains and found that several frailty parameters had significant, age-independent associations with CA severity and a longer course of amyloid disease.

### 4.1. The Frequency of Frailty in CA and a Comparison with HF

We showed in our study that the frequency of frailty was varied by 33% (according to the SEGA), 50% (according to Fried’s measure), or 74% (according to the presence of muscle weakness). Only 8% of patients were robust, according to the physical frailty phenotype. To the best of our knowledge, no frailty-related data on CA have been published previously. However, several studies of patients with HF have shown that the prevalence of frailty varies markedly (from 6.3% to 36%) as a function of the patient profile (acute vs. chronic HF) and the frailty assessment used (i.e., a physical assessment vs. a multidomain assessment) (Supplemental Table S4). We did not compare our data with those from studies that used the frailty index only and did not specify the impaired frailty domains. It is possible that some patients with ATTRwt-CA were not diagnosed and were therefore included in these HF studies—especially when the maintenance of LVEF was an inclusion criteria. Using a multidomain assessment, Madan et al. found a prevalence of frailty of 65%; however, all of the HF patients were NYHA class III or IV and had a low LVEF [44]. Reeves et al. and Vidán et al. reported frailty prevalence of 56% and 74%, respectively; however, these researchers only studied patients with acute decompensated HF [45,46]. Rodrigues Pascual et al. found a prevalence of 57.5%, but their study population was older than ours [47]. Furthermore, 69% of the patients in our study suffered from a loss of autonomy (according to the IADL score), whereas this proportion was only 18.3% and 15% in the other cohorts [48,49]. Similarly, muscle weakness (as assessed by grip strength) was much more common in our population (74%) than in other studies (from 15% to 59%) [45,46,50,51]. Slowness was also more prevalent in our population (48%) than in other studies (from 18% to 36%)—except those that focused on patients with acute decompensated HF [45,46]. Lastly, a risk of depression was more prevalent in our population than in other cohorts [45,48,49].

### 4.2. The Relationship between Frailty and ATTRwt-CA

The frequency of frailty among patients with ATTRwt-CA can be explained by the amyloid fibril infiltration of various organs and tissues—particularly the heart, integumentary system, and nerves. Indeed, muscle weakness could be explained by the high prevalence of carpal tunnel syndrome or Dupuytren’s syndrome, which result from amyloid infiltration [52]. The onset of muscle weakness might be linked to multifactorial neuropathy caused by lumbar spinal stenosis or small fiber neuropathy; in turn, the muscle disorders promote slowness, a loss of mobility, balance disorders, falls, sedentariness, and loss of autonomy. Kharoubi et al. showed that autonomic neuropathy was observed in almost 50% of patients with ATTRwt-CA and was associated with a worse prognosis [53]. Hearing loss is very frequent in ATTRwt-CA [19]. The specific audiologic pattern observed (in which all frequencies are affected) suggests that amyloid fibrils infiltrate the cochlea and/or auditory neural system and thus cause functional impairment [18,19]. Hearing loss may promote social isolation and depression—both of which contribute to frailty [54]. It has already been demonstrated that advanced HF and malnutrition are closely related, as a result of changes in hormone and cytokine levels [55]. The gastroparesis and digestive damage caused by amyloid infiltration might explain the high prevalence of malnutrition and weight loss observed in our study, relative to the literature data studies [56]. However, the relationship between amyloid infiltration and malnutrition must now be documented further by studying body composition. Dysexecutive syndrome and exhaustion were highly prevalent in our study population. In contrast with the literature data [57], we did not observe a significant link between cognitive impairment and the severity of heart failure. In our age-adjusted analyses, only memory complaints were significantly associated with the severity of cardiac amyloidosis. We hypothesis that this lack of an association was, at least in part, due to the small sample size (with an impairment in 12 stage III patients (86%) and 12 stage I and II patients (55%)) and the rather high prevalence of impairment in the seven-point clock drawing test, which explores executive functions (67% of the overall population). Lastly, the large number of comorbidities observed in patients with ATTRwt-CA often results in polypharmacy, which translates into an elevated iatrogenic risk.

Age-independent associations between certain frailty parameters (e.g., balance disorders and a slower gait speed in dual tasks) and a longer course of amyloid disease could be explained by the progression of amyloid infiltration. The severity of balance disorders appears to be associated with greater amyloid deposits in the lumbar spine, in the carpal tunnel, and maybe directly in muscles; this promotes sarcopenia and accelerates the loss of autonomy. Further exploration of this hypothesis might determine whether frailty is a marker of amyloid disease progression and whether treatment ATTR might reduce frailty.

### 4.3. Strengths and Weaknesses

This study’s strength resides in its accurate description of frailty (as assessed with several tools) in elderly patients with ATTRwt-CA. The study’s limitations include its single-center design, the small sample size (limiting the statistical power), and the fact that only one woman was included (limiting external validity). Furthermore, the low proportion of patients having undergone a geriatric assessment might have induced recruitment bias—even though the patient population having agreed to a geriatric assessment and that having refused a geriatric assessment were similar. Lastly, the study lacked a control group of older adults with HFpEF but not ATTR-CA; this might have helped clarify whether the frequency of frailty was truly related to the presence of amyloidosis. These limitations restricted the generalizability of our results. Accordingly, similar analyses should be performed in a larger population.

### 4.4. Clinical Implications: Measurement and Management of Frailty in CA

Our results highlighted several opportunities for improving research on frailty in the setting ATTRwt-CA. At present, there is no gold standard for frailty assessment, and none of the commonly used frailty assessment tools have been specifically validated in patients with CA. We suggest that an assessment of frailty could be incorporated into the routine clinical evaluation of patients with ATTRwt-CA, since it might facilitate risk stratification and treatment decisions. A CGA takes an hour and a half. However, patients who need to be referred to a geriatrician could be screened by a cardiologist using scores (the Fried phenotype and SEGA) that can be rated quickly (in about 10 min).

CGA has improved the management of cancer, predicting toxicity and a decrease in quality of life. It has enabled a more targeted use of preventive measures [58]. In addition, modifiable risk factors such as metabolic syndrome or hyperglycemia increase the cytokine storm in certain cancer cells and cardiomyocytes. This phenomena creates the conditions for cardiotoxicity and immune-resistance [59]. Thus, by preventing these factors, CGA could also generate preventive interest in the management of ATTRwt CA patients.

However, it is not yet clear which interventions can mitigate frailty during the course of amyloid disease. As is already well accepted for HF, it will be important to demonstrate the prognostic impact of frailty in patients with CA, particularly with regard to mortality and hospitalization rates [60,61]. The ATTR-ACT study showed that, at month 30, tafamidis was associated with a significantly (*p* < 0.001) lower rate of decline in distance in the 6-min walk test [23]. Our present findings suggest that other frailty parameters could also be used as outcome measures for CA (SEGA, SPPB, and one-leg standing test). To confirm these observations and to check whether they can be extrapolated, further research should use multidimensional geriatric tools to assess frailty in a larger population of patients with ATTwt-CA and in other subpopulations of patients with CA.

Lastly, it will be important to develop links between geriatricians and cardiologists, with a view of facilitating the diagnosis of frailty, of improving patient care, and of raising awareness of CA among geriatricians.

## 5. Conclusions

Frailty is frequent among patients with ATTRwt-CA. There are significant, age-independent associations between several frailty parameters, and both the severity of CA and the duration of amyloid disease. Patients with ATTRwt-CA should be screened for frailty and managed collaboratively by cardiologists and geriatricians, with the goal of improving quality of life and of assessing the potential value of new (costly) medications.

## Figures and Tables

**Table 1:** Characteristics of the patients with ATTRwt-CA.

Characteristics	N (%)
	*n* = 36
**Demographic characteristics**	
Male sex	35 (97)
Age, years, median (IQR)	82 (76–86)
Living alone	6 (18)
**Cardiology assessment**	
Systolic blood pressure, mmHg, median (IQR)	124 (109–135)
Diastolic blood pressure, mmHg, median (IQR)	76 (68–89)
NYHA class	
I	3 (9)
II	21 (58)
III	12 (33)
IV	0 (0)
Orthostatic hypotension	5 (22)
LVEF, median [IQR]	51 (40–59)
≥45%	22 (62)
<45 %	14 (39)
MCF, %, median (IQR)	25 (19–33)
LVGLS, %, median (IQR)	9.9 (7.8–12.5)
Nt proBNP, ng/L, median (IQR)	3188 (1341–8883)
**Time since the first symptoms of amyloidosis, median (IQR)**	
Extracardiac symptoms *	157 (97–227)
Cardiac symptoms †	49 (15–67)
Cardiac or extracardiac symptoms	146 (73–216)
**Comorbidity assessment**	
**Comorbidities**	
CIRS-G	17 (14–21)
category ≥ 3, median (IQR)	1 (1–2)
Charlson-G, median (IQR)	7 (6–9)
Hypertension	31 (97)
Cancer	3 (9)
Dyslipidemia	17 (51)
Diabetes	6 (18)
Chronic obstructive pulmonary disease	2 (6)
Kidney failure (clearance <60 mL/min CKD-EPI)	20 (56)
Depression	8 (24)
Obesity (body mass index ≥30)	5 (15)
Stroke	5 (15)
Transient ischemic attack	2 (6)
Coronary heart disease	6 (18)
Cognitive disorders	4 (12)
Number of cardiovascular risk factors ≥3 ‡	23 (64)
**Medications taken daily**	
Number of medications, median (IQR)	8 (7–10)
≥5 medications per day	32 (89)
**Frailty scores**	
SEGA	
≤8, not frail	24 (67)
>8 and ≤11, frail	5 (14)
>11, very frail	7 (19)
Physical frailty phenotype (modified CHS criteria) §	
Robust	3 (8)
Pre-frail	15 (42)
Frail	18 (50)

NB: The data are quoted as the frequency (%), unless otherwise stated. Abbreviations: CHS, Cardiovascular Health Study; SEGA, Short Emergency Geriatric Assesment; ATTRwt, wild type transthyretin amyloidosis; NYHA, New York Heart Association; LVEF, left ventricular ejection fraction; LVGLS, left ventricle global longitudinal strain; MCF, myocardial contraction fraction; IQR, interquartile range. * The extracardiac symptoms considered were carpal tunnel syndrome, lumbar canal stenosis, hearing loss, Dupuytren’s syndrome, and peri-orbital bruising; † The cardiac symptoms considered were conduction disorders, atrial fibrillation, dyspnea, edema of the lower limbs, cardiac hypertrophy, chest pain, and aortic stenosis. ‡ The cardiovascular risk factors considered were hypertension, dyslipidemia, diabetes, a family history of cardiovascular disease, and smoking; § The modified CHS criteria were shrinking, self-reported exhaustion, weakness, slowness, and low physical activity (no regular physical activity). Individuals meeting ≥3 criteria are considered to be frail, those meeting 1 or 2 criteria are considered to be pre-frail, and those meeting no criteria are considered to be robust.

**Table 2:** Frailty parameters, according to the Gillmore stage (*n* = 36).

	All	Stage I and II **	Stage III	*p* Value ||	OR (95% CI)	Age-Adjusted	
	(*n* = 36), N (%)	(*n* = 22), N (%)	(*n* = 14), N (%)			*p* Value #	OR [95% CI]
**Demographic caracteristics**							
Male sex	35 (0.97)	21 (95)	14 (100)	1			
Age, years, median [IQR]	82 (76–86)	79.5 (76–82)	86 (80–87)	**0.05**	**1.1 (1.0–1.3)**		
Living alone	6 (18)	4 (19)	2 (17)	1			
**Time since the first amyloidosis symptom, median (IQR)**							
Extracardiac symptoms *	157 (97–227)	169 (122–226)	134 964–227)	0.31			
Cardiac symptoms †	49 (15–67)	43 (16–58)	55 (15–81)	0.67			
Cardiac or extracardiac symptoms	146 (73–216)	163 (108–226)	114 (60–166)	0.1	1.0 (0.9–1.0)	0.12	1.0 (1.0–1.1)
**Frailty scales**							
SEGA, median (IQR)	6 (5–10.5)	5 (4–6)	9 (7–16)	**0.003**	**1.3 (1.1–1.6)**	**0.04**	**1.2 (1.1–1.5)**
SEGA				**0.02**			
≤8, not frail	24 (67)	17 (77)	7 (50)		Ref.		Ref.
>8 and ≤11, frail	5 (14)	4 (18)	1 (7)				
>11, very frail	7 (19)	1 (4)	6 (43)		**2.9 (1.1–7.4)**	0.1	2.3 (0.8–6.4)
Physical frailty phenotype (CHS criteria) ‡				0.23			
Robust	3 (8)	3 (13)	0 (0)				
Pre-frail	15 (42)	10 (45)	5 (36)				
Frail	18 (50)	9 (41)	9 (64)				
**Autonomy and lifestyle**							
ADL < 6	12 (33)	4 (18)	8 (57)	**0.03**	**6.0 (1.3–27)**	**0.05**	**5.0 (1.0–24.0)**
IADL < 8	25 (69)	13 (59)	12 (86)	0.14	4.1 (0.7–23)	0.18	3.4 (0.6–20)
IADL-sf < 4 §	15 (47)	6 (30)	9 (75)	**0.01**	**7 (1.4–35)**	**0.04**	**5.9 (1.1–33)**
Going outside once a week	25(78)	18 (86)	7 (64)	0.2			
Physical and sporting activity	18(54)	12 (57)	6 (50)	0.69			
Hearing impairment	18(50)	12 (55)	6 (43)	0.49			
**Pain on a visual analog scale**	0 (0–2)	0.5 (0–2)	0 (0–2)	0.84			
**Nutrition**							
Risk of malnutrition MNA <12	14(39)	5 (23)	9 (64)	**0.01**	**6.1 (1.4–27)**	**0.09**	**4.3 (0.8–23)**
**Mobility and balance**							
Walks with help	11 (32)	6 (29)	5 (45)	0.44			
Gait speed m/s, median (IQR)	1 (0.7–1)	1 (0.8–1.1)	0.8 (0.6–1)	**0.04**	**0.5 (0.2–1.1)**	0.29	0.2 (0.01–4.3)
Slowness (gait speed <1 m/s)	15 (48)	8 (40)	7 (64)	0.27			
Time taken to walk 10 m in a dual task, s, median (IQR)							
Motor dual task	11 (10–12)	11 (10–11)	11 (10–12.5)	0.31			
Cognitive dual task	12 (10–16)	11 (10–14)	13 (13–16)	**0.05**	**1.3 (0.9–1.7)**	0.31	1.2 (0.9–1.5)
6-min walking test (meters), median (IQR)	322 (247–367)	360 (192–391)	303 (247–330)	0.24			
SPPB, median (IQR)	9 (6–10)	9.5 (8–10)	8 (4–9)	**0.02**	**0.8 (0.6–1.0)**	0.14	0.8 (0.6–1.1)
SPPB				**0.07**			
high (≥10)	13(36)	11 (50)	2 (14)		Ref.		Ref.
moderate (7–9)	13(36)	7 (32)	6 (43)				
low (≤ 6)	10(28)	4 (18)	6 (43)		**2.8 (1.1–7.2)**	**0.09**	**2.4 (0.9–6.3)**
Completion time in a five-time sit-to-stand-test >16.7 s	17 (47)	7 (32)	10 (71)	**0.04**	**5.4 (1.2–23)**	**0.05**	**4.6 (1.0–21)**
Weakness ((grip strength < 30 kg (men) or < 20 kg (women))	23 (74)	14 (67)	9 (90)	0.22			
Non-accidental fall(s) in the past year	15 (42)	7 (32)	8 (57)	0.13	2.9 (0.7–11)	0.3	2.2 (0.5–9.4)
One-leg standing test < 5s	21 (58)	9 (41)	12 (86)	**0.01**	**8.7 (1.5–48)**	**0.04**	**6.4 (1.1–39)**
**Cognitive performance**							
Memory complaints	17 (47)	7 (32)	10 (71)	**0.04**	**5.4 (1.2–23)**	**0.03**	**5.4 (1.1–25)**
MMSE according to age and educational level, median (IQR)	28 (26–29)	28 (27–29)	27 (21–29)	0.11	0.8 (0.6–1.1)		
5-word test score <10	8 (22)	3 (14)	5 (36)	0.22			
7-point clock-drawing test <7	24(67)	12 (55)	12 (86)	**0.08**	**5.0 (0.9–28)**	0.16	3.6 (0.6–22)
Frontal Assessment Battery <16	26(72)	14 (64)	12 (86)	0.26			
Risk of depression, GDS ≥5/15	17(49)	9 (0.43)	8 (0.57)	0.41			
**Sphincter disorders**							
Urinary Symptom Profile							
stress urinary incontinence	0 (0–0)	0 (0–0)	0 (0–1.5)	0.7			
overactive bladder	5 (3–8)	3.5 (2–7)	8 (5.5–9)	**0.01**	**1.7 (1.1–2.7)**	**0.02**	**2.1 (1.1–3.8)**
dysuria	0 (0–1)	0 (0–1)	1 (0.5–2)	**0.04**	**3.1 (1.0–9.8)**	0.17	2.5 (0.7–8.8)
urinary incontinence	8(26)	4 (20)	4 (36)	0.41			

Note. The data are quoted as the frequency (%), unless otherwise stated; Abbreviations: SD, standard deviation; CHS, Cardiovascular Health Study; ADL, Activities of Daily Living; IADL, Instrumental Activities of Daily Living; IADL-sf, Instrumental Activities of Daily Living-short form MMSE, Mini-Mental State Examination; MNA, Mini Nutritional Assessment; GDS, Geriatric Depression Scale; SEGA, Short Emergency Geriatric Assesment; IQR, interquartile range; SPPB, Short Physical Performance Battery. * The extracardiac symptoms considered were carpal tunnel syndrome, lumbar canal stenosis, hearing loss, Dupuytren’s syndrome, and peri-orbital bruising. † The cardiac symptoms considered were conduction disorders, atrial fibrillation, dyspnea, edema of the lower limbs, cardiac hypertrophy, chest pain, and aortic stenosis. ‡ The modified CHS criteria were shrinking, self-reported exhaustion, weakness, slowness, and low physical activity (no regular physical activity). Individuals meeting ≥3 criteria are considered to be frail, those meeting 1 or 2 criteria are considered to be pre-frail, and those meeting no criteria are considered to be robust. § The IADL short form used the “phone”, “treatments”, “money” and “transport” items. || *p* value from the chi squared test, Fisher’s test, Student t-test or non parametric Mann-Whitney’s test, as appropriate. # *p* value from age-adjusted analyses. ** 9 patients (25%) for stage I, and 13 patients (36%) for stage II.

## Data Availability

The data are available upon request depending on patients’ authorization.

## Supplementary Materials

The following are available online at https://www.mdpi.com/article/10.3390/jcm10153415/s1, Table S1: Patient characteristics, Table S2: Cardiac parameters as a function of the Gillmore staging system, Table S3: Relationships between frailty parameters and the time interval between the first symptoms of amyloidosis and the geriatric assessment, Table S4: Characteristics of studies of frailty and HF
