# Supplementary material for: Frailty in Wild-Type Transthyretin Cardiac Amyloidosis: The Tip of the Iceberg
Source: J Clin Med. 2021 Jul 31;10(15):3415. doi: 10.3390/jcm10153415 (PMC8348590; doi:10.3390/jcm10153415)
Supplement: Supplementary file 1 [file jcm-10-03415-s001.zip › jcm-1308472-supplementary.pdf]

SUPPLEMENTAL MATERIAL

Supplemental Table S1. Patient characteristics

<u>Characteristics</u>	Geriatric assesment<br><br>n=36	Refused or did not attend a geriatric assessment<br><br>n=144	P value*
Male sex	35 (97)	122 (85)	0.05
Age, years, median [IQR]	82 [76-86]	80 [75-85]	0.35
NYHA class			0.6
I	3 (9)	17 (12)	
II	21 (58)	75 (54)	
III	12 (33)	41 (29)	
IV	0 (0)	7 (5)	
LVEF, median [IQR]			0.68
≥ 45%	22 (61)	83 (65)	
< 45 %	14 (39)	45 (35)	
LVGLS, median [IQR]	9.9 [7.8-12.5]	9.7 [7.4-12.9]	0.79
Nt proBNP, ng/l, median [IQR]	3188 [1341-8883]	3069 [1303-4863]	0.23

NB: the data are quoted as the frequency (%), unless otherwise stated.

Abbreviations: NYHA, New York Heart Association ; LVEF, left ventricular ejection fraction; LVGLS, left ventricle global longitudinal strain; IQR, interquartile range

* P value from the chi squared test, Fisher's test, Student t-test or non parametric Mann-Whitney's test, as appropriate

Supplemental Table S2 . Cardiac parameters, as a function of the Gillmore staging system (n=36)			
	**Stage I and II*** **(n=22), N (%)**	**Stage III** **(n=14), N (%)**	**P value †**
Systolic blood pressure, mmHg, median [IQR]	123 [117-129]	118 [111-122]	0.6
Diastolic blood pressure, mmHg, median [IQR]	68 [64-70]	70[68-81]	0.2
NYHA class			**0.02**
I	0 (0)	3 (21)	
II	16 (73)	5 (36)	
III	6 (27)	6 (43)	
IV	0 (0)	0 (0)	
LVEF, median [IQR]	53.5 [47-58]	40 [35-60]	**0.04**
≥ 45%	17 (77)	5 (36)	**0.01**
< 45 %	5 (23)	9(64)	
LVGLS, median [IQR]	10.2 [8.3-12.4]	8.8 [7.2-12.5]	0.3
MCF, median [IQR]	27 [20-33]	19 [13-26]	**0.04**
Nt proBNP, ng/l, median [IQR]	1712 [754-2944]	8883 [5902-13122]	**<0.0001**

NB: The data are quoted as the frequency (%), unless otherwise stated.

Abbreviations: NYHA, New York Heart Association ; LVEF, left ventricular ejection fraction; LVGLS, left ventricle global longitudinal strain; MCF, Myocardial contraction fraction; IQR, interquartile range

*9 patients (25%) for stage I, and 13 patients (36%) for stage II

† P value from the chi squared test, Fisher's test, Student t-test or non parametric Mann-Whitney's test, as appropriate

Supplemental Table S3. Relationships between frailty parameters and the time interval between the first symptoms of amyloidosis and the geriatric assessment (n=36)

**Frailty parameters from the geriatric assessment**		**N**	**Time since the first symptoms of amyloidosis Median [IQR]**	***P* value ***	***P* value †**
**Frailty scales**					
SEGA	>8, frail and very frail	9	226 [158-240]	**0.09**	**0.09**
	≤ 8 , not frail	23	145 [64-184]		
Physical frailty phenotype (modified CHS criteria) ‡	Frail	15	157 [86-240]	0.37	0.21
	Non-frail	17	157 [108-184]		
**Autonomy and lifestyle**					
ADL			Rho=-0.2	0.27	1
	<6	8	196 [115-283]	0.28	0.17
	≥ 6	24	146 [97-194]		
IADL			Rho=-0.19	0.26	0.23
	<8	21	165 [86-227]	0.56	0.49
	≥ 8	11	145 [122-170]		
IADL-sf §			Rho=-0.27	0.22	0.39
	<4	12	226 [103-258]	0.1	**0.09**
	≥ 4	17	145 [108-170]		
**Pain on a visual analog scale**			Rh=-0.06	0.72	0.45
**Risk of malnutrition (MNA)**	MNA <12	11	166 [72-240]	0.59	0.38
	MNA ≥ 12	21	148 [122-184]		
**Mobility and balance**					
Walks with help	yes	9	226 [157-289]	**0.02**	**0.03**
	No	21	145 [86 - 181]		
6-minute walk distance, m			Rho=-0.13	0.54	0.11
Gait speed			Rh=-0.32	**0.09**	0.1
	<1 m/s	13	181 [145-240]	0.23	0.18
	≥1 m/s	16	146 [115-194]		
Time to walk 10 meters in a dual task, s					
	Cognitive dual task		Rho=0.3	0.12	**0.008**
	Motor dual task		Rho=0.28	0.17	**0.05**
SPPB			Rho=-0.25	0.41	0.35
	high physical performance (≥10)	13	144 [108-170]	0.34	Ref.
	moderate physical performance (7-9)	13	166 [134-240]		0.11
	low physical performance (≤ 6)	6	192 [86-227]		0.26
Completion time in a five-time sit-to-stand-test, seconds	>16,7	13	226 [134-240]	**0.07**	**0.06**
	≤ 16,7	19	145 [64-181]		
weakness (grip strength < 30 kg (males), < 20 kg (females))	Yes	20	152 [120-228]	0.84	0.59
	No	8	177 [86-215]		
Non-accidental fall(s) in the past year	yes	12	169 [90-251]	0.51	0.26
	No	20	152 [104-195]		
One-leg standing test < 5s	Yes	17	145 [72-229]	0.8	0.82
	No	15	165 [122-184]		
**Cognitive performance**					
MMSE, according to age and educational level			Rho=-0.15	0.41	0.65
5-word test score <10	Yes	5	122 [51-166]	0.14	0.16
	No	27	157 [108-229]		
7-point clock-drawing test <7	Yes	20	157 [90-226]	0.64	0.79
	No	12	164 [109-240]		
Frontal Assessment Battery <16	Yes	10	168 [122-229]	0.18	0.4
	No	22	145 [64-169]		
Risk of depression, GDS ≥ 5/15	Yes	16	157 [86-226]	0.78	0.69
	No	15	170 [115-228]		

Note. The data are quoted as the median [interquartile range], unless otherwise stated

Abbreviations: CHS, Cardiovascular Health Study; ADL, Activities of Daily Living; IADL, Instrumental Activities of Daily Living; IADL-sf, Instrumental Activities of Daily Living-short form MMSE, Mini-Mental State Examination; MNA, Mini Nutritional Assessment; GDS, Geriatric Depression Scale ; SEGA, Short Emergency Geriatric Assesment; IQR, interquartile range; SPPB, Short Physical Performance Battery

* P value from the non-parametric Mann-Whitney test, the Kruskal Wallis test or Spearman's rank correlation test, as appropriate

† P value from age-adjusted analyses using multivariate logistic or quantile regression models

‡ The modified CHS criteria were shrinking, self-reported exhaustion, weakness, slowness, and low physical activity (no regular physical activity). Individuals with ≥3 criteria are considered to be frail, those with 1–2 criteria are considered to be pre-frail, and those with no criteria are considered to be robust.

§ The IADL short form used the "phone", "treatments", "money" and "transport" items

Supplemental Table S4. Characteristics of studies of frailty and HF

First Author (year, country)	Age mean ± SD or median [IQR]	Male %	Sample Size	Type of patient	LVEF (mean ± SD) or median [IQR] or % LVEF≤45%)	NYHA class III-IV (%)	**Fried phenotype** : Frails	Abnormal OARS* or IADL**	Risk of malnutrition **MNA** <12	**Muscle weakness** ((grip strength < 30 kg (men) or < 20 kg (women))	**SPPB**<10	**Slowness** (gait speed <1 m/s)	**Risk of depression (GDS**sf ≥1 or **GDS** ≥ 5 out of 15)
**Broussier (2021, France)***	**82 (76-86)**	**97**	**36**	**ATTRwt-CA in outpatients and inpatients**	50.5 (40-59)	**33**	**50%**	**69% (5.3 ± 2.4) ****	**39%**	**74%**	**64% (36% SPPB≤8, 28% SPPB 0-4)**	**48% (19% <0.65m/s, 35% <0.8 m/s)**	**49%**
Boxer (2010, USA)	77 ± 10	72	60	CHF with LVEF ≤ 40%	EF≤ 40%	42.4	25%	-	-	-	-	-	-
Martin Sanchez (2017, Spain)	82.4 ± 7.2	39	465	Acute HF	58.3% (LVEF ≤45%)	23.1	36%	-	-	43%	-	36%	-
McNallan (2013, USA)	73.2 ± 13.3	57.4	448	Inpatients and outpatients with HF	45 ± 16.3	-	19%	-	-	-	-	-	-
Newman (2001, USA)	71.5 ± 4.6	-	4735	CHF outpatients	-	-	6.3 %	-	-	-	-	-	-
Nishiguchi (2016, Japan)	73.7±7.3	69.4	206	HF outpatients		9.3	16.5 %	-	-	15%	-	18%	-
Reeves (2016, USA)	72 ±10	41	27	ADHF	37 ±16	-	56%	-	-	56%	56% (SPPB<6),	74%	30%
Vidan (2016, Spain)	80.0 ± 6.1	50.5	416	CHF inpatients. HFpEF (79.3%)	53% (LVEF ≤50%)	25.5	74%	-	-	59%	-	74%	-
Rodriguez-Pascual (2017, Spain)	85.2 ±7.3	39	497	CHF. HFpEF (79.3%)	20.7% (LVEF ≤45%)	27.9	57.5 %	-	-	-	-	-	-
Madan (2016, USA)	74.9 ± 6.5	42.5	40	CHF. HFrEF (100%)	25.6 ± 6.4%	100	65%	-	-	-	-	-	-
Pulignano (2016, Italy)	77.4 ± 5.9	57	331	CHF. HFpEF (19.9%)	34.6 ± 11.6	51.4	-	-	-	-	-	34.7% (<0.65 m/s)	-
Chaudhry (2013, USA)	79.7 ± 6.2	49.5	758	Newly diagnosed HF. HFpEF (56.8%)	43.2% (LVEF ≤45%)	30.3	-	-	-	41.8%	-	41.8% (<0.8 m/s)	-
Chiarantini (2010, Italy)	80.0 ± 13.8	50.3	157	AHF. HFpEF (46%)	54% (LVEF ≤45%)	59.9	-	-	-	-	50.9% (SPPB 0-4)	-	-
Altimir (2005, Spain)	65.2 ± 10.9	72.5	360	Outpatients with HF	31.7 ± 12.6	48.3	-	18.3%**	-	-	-	-	29.7 %
Pilotto (2010, Italy)	80.5 ±7.3	-	376	HF	47.9 ± 13	-	-	3.4 ± 2.7 *	21.5±4.5	-	-	-	-

Abbreviations: AHF, acute heart failure ; HFpEF, heart failure with preserved ejection fraction; HFrEF, heart failure with reduced ejection fraction; MGA, Multidimensionnal Geriatric Assessment; CHF, chronic heart failure; SD, standard deviation; SEGA, Short Emergency Geriatric Assessment ; ATTRwt, wild type transthyretin amyloidosis ; NYHA, New York Heart Association; NR, not reported; LVEF, left ventricular ejection fraction; LVGLS, left ventricle global longitudinal strain; IQR, interquartile range ; SPPB, Short Physical Performance Battery

* All patients had ATTRwt amyloidosis. According to the SEGA, 33% had some degree of frailty (14% were frail and 19% were very frail)
